# RhoGEF Trio Regulates Radial Migration of Projection Neurons *via* Its Distinct Domains

**DOI:** 10.1007/s12264-021-00804-7

**Published:** 2021-12-16

**Authors:** Chengwen Wei, Mengwen Sun, Xiaoxuan Sun, Hu Meng, Qiongwei Li, Kai Gao, Weihua Yue, Lifang Wang, Dai Zhang, Jun Li

**Affiliations:** 1 Peking University Sixth Hospital, Peking University Institute of Mental Health, NHC Key Laboratory of Mental Health (Peking University), National Clinical Research Center for Mental Disorders (Peking University Sixth Hospital), Beijing, 100191 China; 2 Peking-Tsinghua Center for Life Sciences, Peking University, Beijing, 100871 China; 3 PKU-IDG/McGovern Institute for Brain Research, Peking University, Beijing, 100871 China; 4 Chinese Institute for Brain Research, Beijing, 100010 China; 5 Institute for Brain Research and Rehabilitation (IBRR), Guangdong Key Laboratory of Mental Health and Cognitive Science, South China Normal University, Guangzhou, 510631 China

**Keywords:** *Trio*, Myosin X, RhoGEF, Neurodevelopmental disorder, Radial migration

## Abstract

The radial migration of cortical pyramidal neurons (PNs) during corticogenesis is necessary for establishing a multilayered cerebral cortex. Neuronal migration defects are considered a critical etiology of neurodevelopmental disorders, including autism spectrum disorders (ASDs), schizophrenia, epilepsy, and intellectual disability (ID). *TRIO* is a high-risk candidate gene for ASDs and ID. However, its role in embryonic radial migration and the etiology of ASDs and ID are not fully understood. In this study, we found that the *in vivo* conditional knockout or *in utero* knockout of *Trio* in excitatory precursors in the neocortex caused aberrant polarity and halted the migration of late-born PNs. Further investigation of the underlying mechanism revealed that the interaction of the Trio N-terminal SH3 domain with Myosin X mediated the adherence of migrating neurons to radial glial fibers through regulating the membrane location of neuronal cadherin (N-cadherin). Also, independent or synergistic overexpression of RAC1 and RHOA showed different phenotypic recoveries of the abnormal neuronal migration by affecting the morphological transition and/or the glial fiber-dependent locomotion. Taken together, our findings clarify a novel mechanism of Trio in regulating N-cadherin cell surface expression *via* the interaction of Myosin X with its N-terminal SH3 domain. These results suggest the vital roles of the guanine nucleotide exchange factor 1 (GEF1) and GEF2 domains in regulating radial migration by activating their Rho GTPase effectors in both distinct and cooperative manners, which might be associated with the abnormal phenotypes in neurodevelopmental disorders.

**Supplementary Information:**

The online version contains supplementary material available at 10.1007/s12264-021-00804-7.

## Introduction

During neocortical development, the highly organized layering is achieved *via* the radial migration of neurons from the ventricular zone (VZ) to the destined layers in an inside-out manner [1]. The precursors of pyramidal neurons (PNs) undergo a multipolar (MP) to bipolar (BP) morphological transition as they migrate from the lower to the upper intermediate zone (IZ). BP PNs migrate radially into the cortical plate (CP) through glial-guided locomotion, then undergo somal translocation to reach their final position, where they are wired into functional circuits [2, 3]. Neuronal migration deficits are considered to be an important etiology of many neurodevelopmental disorders, including autism spectrum disorders (ASDs), schizophrenia, epilepsy, and intellectual disability (ID) [4–8]. However, the mechanisms underlying neuronal migration deficits in the etiology of these diseases remain unclear.

*TRIO* (trio Rho guanine nucleotide exchange factor) encodes a guanine nucleotide exchange factor (GEF) that facilitates the activation of Rho GTPases. Previous studies mainly focused on the roles of Trio in postmitotic PNs, including axon guidance [9, 10], dendritic branching, and synaptic transmission and plasticity [11, 12]. Although the olfactory bulb, hippocampus, and cerebellum develop abnormally upon *Trio* deletion [13, 14], the role of *Trio* in the embryonic development of the neocortex, especially in the radial migration of PNs, is unclear. The contribution of Rho GEFs to the pathogenesis of neuropsychiatric disorders, including ASDs, was recently reported [15]. Moreover, a cluster of deleterious *de novo* mutations in TRIO GEF1 and the spectrin repeat domain were recently identified in neurodevelopmental disorders including ASDs and/or ID [16–18], suggesting that Trio dysfunction is a risk factor for neurodevelopmental diseases. However, little attention has been devoted to the involvement of *Trio*-mediated cortical radial migration in the etiology of ASDs and ID. Furthermore, Trio contains two independent GEF domains for the specific activation of Rac1/RhoG (GEF1/N-terminal GEF domain) and RhoA (GEF2/C-terminal GEF domain) [19–21]. Both the DH1 and PH1 domains in GEF1 play a role in Rac1 interaction and activation as previously reported [22]. The N-terminal SH3 has been reported to modulate GEF1 activity but is not required for Rac1 or RhoG guanosine diphosphate (GDP)-guanosine triphosphate (GTP) exchange [23, 24]. However, the roles of different domains of *Trio* in embryonic radial migration are largely unknown and need further clarification.

In this study, the function of *Trio* in cortical migration was addressed *in vivo* through the deletion of *Trio* in excitatory progenitors using *in utero* electroporation of Cre recombinase. The morphogenesis and migratory behaviors of the *Trio*-ablated neurons were investigated by immunofluorescence against various markers. Trio promoted neuronal morphogenesis during the MP-to-BP transition in the IZ and the extension of the leading process of late-born neurons. In addition, the interaction of Trio N-terminal SH3 with Myosin X was essential for radial glial-dependent migration through regulating the neuron‐glia adhesion mediated by the membrane location of N-cadherin in the CP. Trio promoted the radial migration *via* its GEF1 and GEF2 domains in the different Rho GTPase signaling pathways. Our study on the function of Trio in embryonic radial migration provides new insights into the involvement of neuronal migration in the pathogenesis of neurodevelopment disorders.

## Materials and Methods

### Animals

*Trio*^*fl/fl*^ mice were purchased from the Model Animal Research Center (Nanjing, China) as previously described [14] and were backcrossed with C57BL/6 mice for >10 generations. For *in utero* electroporation, the *Trio*^*fl/fl*^ C57/BL6 mice were crossed with ICR mice to obtain a *Trio*^*fl/fl*^ inbred ICR strain. Emx1-Cre mice were crossed with *Trio* ^*fl/fl*^ mice to generate *Trio*^*fl/fl:Emx-Cre*^ mice. All animal experiments were performed in strict accordance with the Peking University institutional animal care and use guidelines and approved by the Animal Care Committee.

### Antibodies

The primary antibodies used were as follows: rabbit anti-Trio (Santa Cruz, sc-28564, 1:50), rabbit anti-GFP (Invitrogen, A1112, 1:2000), mouse anti-GFP (Invitrogen, MA5-15256, 1:200), mouse anti-Nestin (Millipore, MAB353, 1:100), rabbit anti-GRASP65 (Abcam, ab174834, 1:1000), rabbit anti-N-cadherin (Cell Signaling Technology, 3116s, 1:100), rabbit anti-Ki67 (Abcam, AB15580, 1:500), mouse anti-Tuj1 (Sigma–Aldrich, T8660, 1:1000), rabbit anti-Cux1 (Santa Cruz, sc-13024, 1:200), rabbit anti-Tbr1 (Abcam, ab31940, 1:1000), rabbit anti-caspase-3 (Cell Signaling Technology, P42574, 1:500), mouse anti-Flag (Sigma–Aldrich, F3165, 1:2000), rabbit anti-HA (Sigma–Aldrich, 05-902R, 1:2000), and rabbit anti-GAPDH (Cell Signaling Technology, 5174, 1:2000). F-actin was stained with Alexa-555 phalloidin (Invitrogen, A34055, 1:100). The secondary antibodies used were as follows: Alexa 488 anti-rabbit (Invitrogen, A11008, 1:1000), Alexa 555 anti-rabbit (Invitrogen, A21428, 1:1000), Alexa 488 anti-mouse (Invitrogen, A11001, 1:1000), and Alexa 555 anti-mouse (Invitrogen, A21422, 1:1000).

### Plasmid Construction

The following constructs were used for *in utero* electroporation and transfection in primary cultured neurons: *pcAGGS-Cre-HA-IRES-EGFP*, *pNeuroD1-Cre-IRES-EGFP*,* pcAGGS-MYOSIN X-3Flag-IRES-EGFP*, *pcDNA3.1-TRIO9S-HA*, *pcDNA3.1-TRIO9S-ΔSP-HA*, *pcDNA3.1-TRIO9S-ΔGEF1-SH3-HA*, *pcDNA3.1-TRIO9S-ΔGEF1-HA*, *pcDNA3.1-TRIO9S-ΔGEF2-HA*, *pcAGGS-RHOA-IRES-EGFP*, *pcAGGS-RAC1-IRES-EGFP*, *pcAGGS-CA[*constitutively *active (CA)]-PAK1-HA-IRES-EGFP*, and *pcAGGS-CA-ROCK-HA-IRES-EGFP*. The human Myc-TRIO-FL plasmid was a gift from Prof. Richard E. Mains (University of Connecticut Health Center, MI, USA). The TRIO9S-HA plasmid was constructed based on the Myc-Trio-FL plasmid. The TRIO9S-ΔGEF1-SH3-HA, TRIO9S-ΔGEF1-HA, TRIO9S-ΔGEF2-HA, and TRIO9S-ΔSP-HA plasmids were further constructed to delete the GEF1-SH3, GEF1, GEF2, and SP domains based on the TRIO9S-HA plasmid, respectively. Human MYOSIN X, RAC1, RHOA, and CA mutants of PAK1 and ROCK were amplified from cDNA and cloned into the *pcAGGS-IRES-EGFP* plasmid.

### Co-immunoprecipitation and Immunoblotting

HEK293T cells were cultured in Dulbecco’s modified Eagle’s medium (DMEM) supplemented with 10% fetal bovine serum (FBS), 100 U/mL penicillin, and 100 mg/mL streptomycin, transfected with appropriate expression plasmids using jetOPTIMUS (Polyplus-transfection, France), and incubated in a humidified 5% CO_2_ atmosphere at 37°C for 48 h. The cells were harvested in phosphate-buffered saline (PBS), spun down, and re-suspended on ice with lysis buffer [50 mmol/L Tris/HCl (pH 8.0), 200 mmol/L NaCl, 1% Triton X-100, complete proteinase inhibitors (Roche), and EDTA]. The proteins were solubilized by rotating the cell lysates at 4°C for 20 min on a rotating wheel, and the supernatants were then cleared by centrifugation at 12,000 *g* for 15 min at 4°C. The supernatant lysate was incubated with 2 µg anti-HA or anti-FLAG co-injected beads overnight at 4°C. The beads were washed four times with the lysis buffer. The bound proteins were eluted with 2× Laemmli sample buffer (Sigma–Aldrich) and loaded onto sodium dodecyl sulfate‐polyacrylamide gel electrophoresis (SDS-PAGE) for immunoblotting using rabbit anti-HA (1:2000) and mouse anti-Flag (1:2000). For brain tissue, the E14.5 cortex was homogenized with RIPA [50 mmol/L Tris (pH 7.4), 150 mmol/L NaCl, 1% Triton X-100, and 0.1% SDS] supplemented with protease inhibitors (Roche). The lysates were centrifuged at 12,000 *g* for 20 min at 4°C. The supernatants were collected and boiled with 2× Laemmli sample buffer (Sigma–Aldrich) and loaded onto SDS-PAGE. After membrane transfer, rabbit anti-Trio (1:500), mouse anti-GAPDH (1:2000), and HRP-conjugated secondary antibodies (1:1000) were used for immunoblotting. The membranes were developed and imaged using the enhanced chemiluminescence reagent (Thermo Fisher Scientific). To quantify the intensity of bands of interest, three independent experiments for each molecule were analyzed using ImageJ software (NIH). GAPDH was used as an internal control to normalize band intensity.

### Immunofluorescence

Pregnant mice were anesthetized, and the embryos were perfused with 4% (*w*/*v*) paraformaldehyde (Sigma–Aldrich) in PBS. The brains were removed and post-fixed overnight at 4°C, following dehydration in 25% (*w*/*v*) sucrose diluted in PBS for 2 days at 4°C. The brains were embedded an optimum cutting temperature compound and sectioned coronally at 25 μm on a cryostat (Leica Biosystems, Germany). The sections were treated with citrate antigen retrieval solution (Beyotime, China) at 80°C for 12 min if necessary. After three times washing with 1× PBS for 10 min each time, the sections were blocked with 5% (*w*/*v*) bovine serum albumin and 0.1% Triton X-100 in PBS for 30 min at room temperature and incubated at 4°C with the indicated primary antibodies for 18 h. The sections were then rinsed three times with 0.01% Triton X-100 in PBS for 10 min each and incubated with Alexa Flour 488 and 555 for 1 h at room temperature. The nuclei were stained using Hoechst 33342 (Invitrogen) at room temperature for 10 min. The sections were imaged with an FV-1200 confocal laser scanning biological microscope (Olympus, Japan) and an IX71 inverted fluorescence microscope (Olympus, Japan).

### *In Utero* Electroporation

Pregnant mice were deeply anesthetized, and the intrauterine embryos were surgically manipulated as described previously [25]. In brief, *pcAGGS* carrying *Cre* and internal ribozyme entry site (*IRES*) driving enhanced green fluorescent protein (*EGFP*) was purified without endotoxin. The concentration of the plasmid was adjusted to 2 mg/mL. The plasmid containing 0.02% Fast Green solution was injected into the lateral ventricle of embryos at the indicated time. The plasmids were delivered into the VZ surface of the CP in the somatosensory cortical region by electronic pulses (Nepa Gene, Japan). The operated embryos were allowed to live within the uterine horn until the time of observation.

### Brain Slice Culture

For embryonic brain slice culture, the brains were dissected out in cold Hibernate-E medium (Gibco). Brain slices (300 μm thick) from E15.5 embryos electroporated with the indicated plasmids at E13.5 were prepared with a Leica Vibratome VT1200. To visualize neuronal migration, the slices were transferred onto Millicell inserts (Millipore) in Neurobasal medium (Gibco) containing 20% FBS, 2 mmol/L L-glutamine, and penicillin/streptomycin (50 U/50 μg/mL). The glass-bottomed dish was then fitted into a temperature-controlled chamber on the microscope stage and incubated for 16 h at 37°C under a 5% CO_2_ air atmosphere. Live cell images were captured using an Olympus FV1200 Viewer laser scanning confocal microscope.

### Primary Neuronal Culture

The cortical tissue from E16.5 embryos electroporated with the indicated plasmids at E14.5 were digested with 0.125% trypsin at 37°C for 15 min and dissociated by pipetting in DMEM with 20% FBS. The medium was changed to Neurobasal medium (Gibco) with 2% B27 supplement (Gibco) and 2 mmol/L L-glutamine (Gibco) 2 h after seeding.

## Results

### Trio Regulates Radial Migration and Leading Process Morphology of Late-born Neurons in the Embryonic Cortex

**Fig. 1:**
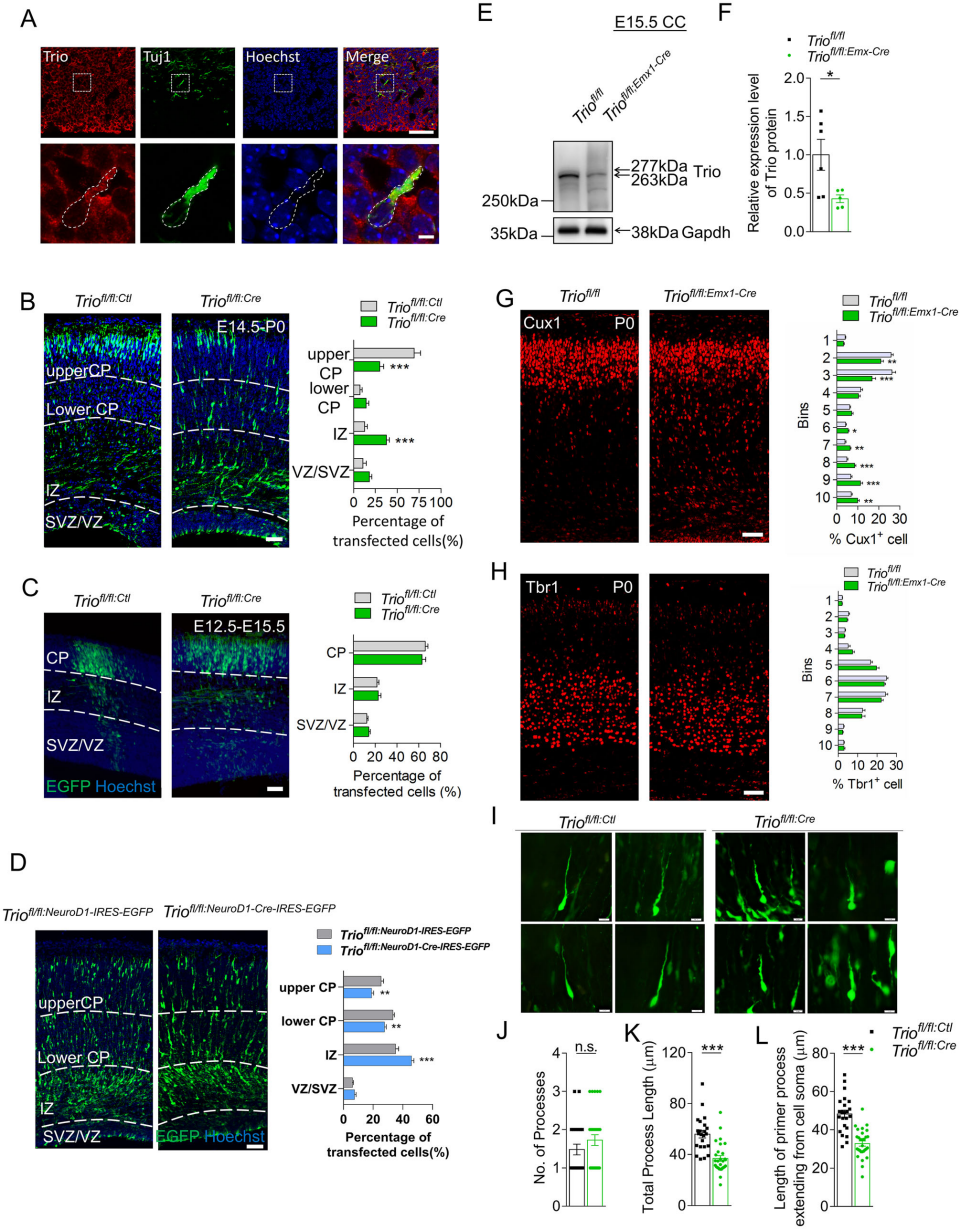
Depletion of Trio impairs embryonic radial migration of projection neurons. **A** High expression of Trio in postmitotic neurons. Tuj1 and Trio are co-labeled in the developing neurons of the ventricular and subventricular zones at E14.5. The dashed boxes in the upper panels are magnified in the lower panels. Nuclei stained with Hoechst. Scale bars, 50 μm (upper panels) and 5 μm (lower panels). **B, C** Left panels, endogenous Trio is knocked out by *in utero* electroporation (IUE) at E14.5 (**B**) and E12.5 (**C**) with overexpression of the *pcAGGS-Cre-IRES-EGFP* plasmid in the progenitors of *floxed-Trio* mice using the *pcAGGS-IRES-EGFP* plasmid as a control. Nuclei stained with Hoechst to mark distinct CP, IZ, and SVZ/VZ layers. Scale bars, 50 μm. Right panels, the distribution and frequency of cortical neurons at P0 (**B**) and E15.5 (**C**) after electroporation (*n* = 12 slices from 3 *Trio*^*fl/fl:Ctl*^ mice and *n* = 12 slices from 3 *Trio*^*fl/fl:Cre*^ mice). **D** Left panels, endogenous Trio in postmitotic neurons is knocked out by IUE at E14.5 with the *pNeuroD1-Cre-IRES-EGFP* plasmid in *floxed-Trio* mice using the *pNeuroD1-IRES-EGFP* plasmid as a control. Distinct CP, IZ, and SVZ/VZ layers are marked. Scale bar, 50 μm. Right panel, the distribution and frequency of the cortical neurons at E18.5 (*n* = 12 slices from 3 *Trio*^*fl/fl:NeuroD1-IRES-EGFP*^ mice and *n* = 12 slices from 3 *Trio*^*fl/fl:NeuroD1-Cre-IRES-EGFP*^* mice*). **E, F** Reduced protein level of Trio in the *Trio*^*fl/fl:Emx1-Cre*^ cortical cortex. Western blots at E15.5 showing Trio protein expression in *Trio*^*fl/fl:Emx1-Cre*^ and *Trio*^*fl/fl*^ cortex using the Trio antibody; GAPDH was used as an internal reference (**E**). Relative Trio protein levels in the two groups (**F**) (*n* = 5 *Trio*^*fl/fl*^ mice and *n* = 5 *Trio*^*fl/fl:Emx1-Cre*^ mice). **G, H** Left panels, cortical sections of P0 *Trio*^*fl/fl:Emx1-Cre*^ and *Trio*^*fl/fl*^ mice immunostained using Cux1 and Tbr1 antibodies. Scale bar, 50 μm. Right panels, changes in the distribution of Cux1^+^ and Tbr1^+^ cells across 10 equal bins in the two groups. **I–L** Higher magnification images showing the morphology of the neuronal leading process in *Trio*^*fl/fl:Cre*^ and *Trio*^*fl/fl:Ctl*^ neurons (**I**). Scale bar, 10 μm. Statistical analysis of the number of processes (**J**), total process length (**K**), and length of a primary process extending from the soma (**L**) (*n* = 23 *Trio*^*fl/fl:Ctl*^ neurons and *n* = 29 *Trio*^*fl/fl:Cre*^ neurons). Histograms show the mean ± SEM; *P < 0.05; **P < 0.01; ***P < 0.001, Student’s *t* test.

We first examined the expression of Trio in the developing cortex by immunostaining to explore its role in cortical morphogenesis. Trio was co-localized with Tuj1, a β-tubulin marker specific to postmitotic neurons in the embryonic cortex (Fig. 1A), indicating that Trio might play a critical role in the cortical development. We then investigated Trio function in projection neurons during neocortical development. Progenitors in the VZ of *Trio*^*fl/fl*^ embryos were electroporated with the chicken beta‐actin (*CAG*) promoter‐driven *Cre-IRES-EGFP* plasmid to delete *Trio* in early-born (E12.5) and late-born (E14.5) neurons. The *CAG* promoter‐driven *IRES-EGFP* plasmid was used as a control. In Trio-deleted embryos, EGFP-positive (EGFP^+^) late-born cortical neurons abnormally accumulated in the IZ and fewer neurons were found in the upper CP at P0 (Fig. 1B). Trio ablation had no effect on the radial migration of early-born projection neurons (Fig. 1C). To exclude the possibility that the impaired radial migration was due to the effect of *Trio* deletion on progenitor proliferation and/or cell-cycle exit, the *pNeuroD1-Cre-IRES-EGFP* vector was designed and generated to allow Cre expression in early postmitotic neurons under the control of the mouse *NeuroD1* promoter [26]. The E14.5 electroporated *Trio*^*fl/fl:NeuroD1-Cre-IRES-EGFP*^ neurons showed defective migration relative to the *Trio*^*fl/fl:NeuroD1-IRES-EGFP*^ neurons at E18.5 (Fig. 1D). *Trio*^*fl/fl:Emx1-Cre*^ mice were generated by crossing *Trio*^*fl/fl*^ mice with the *Emx1-Cre* line to delete Trio in the forebrain excitatory progenitors so as to further confirm the abnormal migration of neurons in the neocortex. Trio protein levels decreased significantly in the embryonic cortex of *Trio*^*fl/fl:Emx1-Cre*^ mice compared with controls (Fig. 1E, F). The neocortex was immunostained for Cut like homeobox 1 (Cux1) and T-box brain transcription factor 1 (Tbr1), markers of neurons from cortical layers II–IV and VI, respectively (Fig. 1G, H). Consistent with the abnormal migration phenotypes of neurons labeled using electroporation, Cux1-positive (Cux1^+^) cells accumulated in the IZ of *Trio*^*fl/fl:Emx1-Cre*^ mice at P0, leading to a lower proportion of neurons in the CP relative to the amounts found in control (Fig. 1G). In addition, the distribution of Tbr1-positive (Tbr1^+^) neurons was not significantly different between the two genotypes (Fig. 1H). The total length of the leading process and the length of the primary process extending from the soma were significantly shorter in the electroporated *Trio*^*fl/fl:Cre*^ neurons than in *Trio*^*fl/fl:Ctl*^ neurons, while the number of processes was similar (Fig. 1I–L). In addition, the *Trio*^*fl/fl:Cre*^ and *Trio*^*fl/fl:Ctl*^ neurons electroporated at E14.5 were also analyzed at P4. Trio-deleted neurons exhibited sustained location defects, as many more migratory EGFP^+^ cells were localized in layers V-VI compared with the control neurons. This was consistent with the arrested neurons in the IZ during embryogenesis. A portion of the ectopic *Trio*^*fl/fl:Cre*^ neurons was localized in the subcortical white matter (WM) (Fig. S1A). Morphological analysis showed abnormal leading processes and stunted neurites in Trio-depleted neurons in layers II-IV compared with controls (Fig. S1A). The extensions of axons from electroporated ipsilateral neurons to the contralateral side were shorter for *Trio*^*fl/fl:Cre*^ neurons than for controls at P4 (Fig. S1B). Furthermore, the ectopic neurons present in the WM were positive for the layer II–IV marker Cux1, but negative for the layer VI marker Tbr1 (Fig. S1C). Altogether, these results suggested that *Trio* deletion impairs the morphogenesis and migration of late-born cortical projection neurons at embryonic and early postnatal development stages.

### *Trio*-deleted Projection Neurons Exhibit an Aberrant Multipolar-to-Bipolar Transition and Migration Velocity

**Fig. 2:**
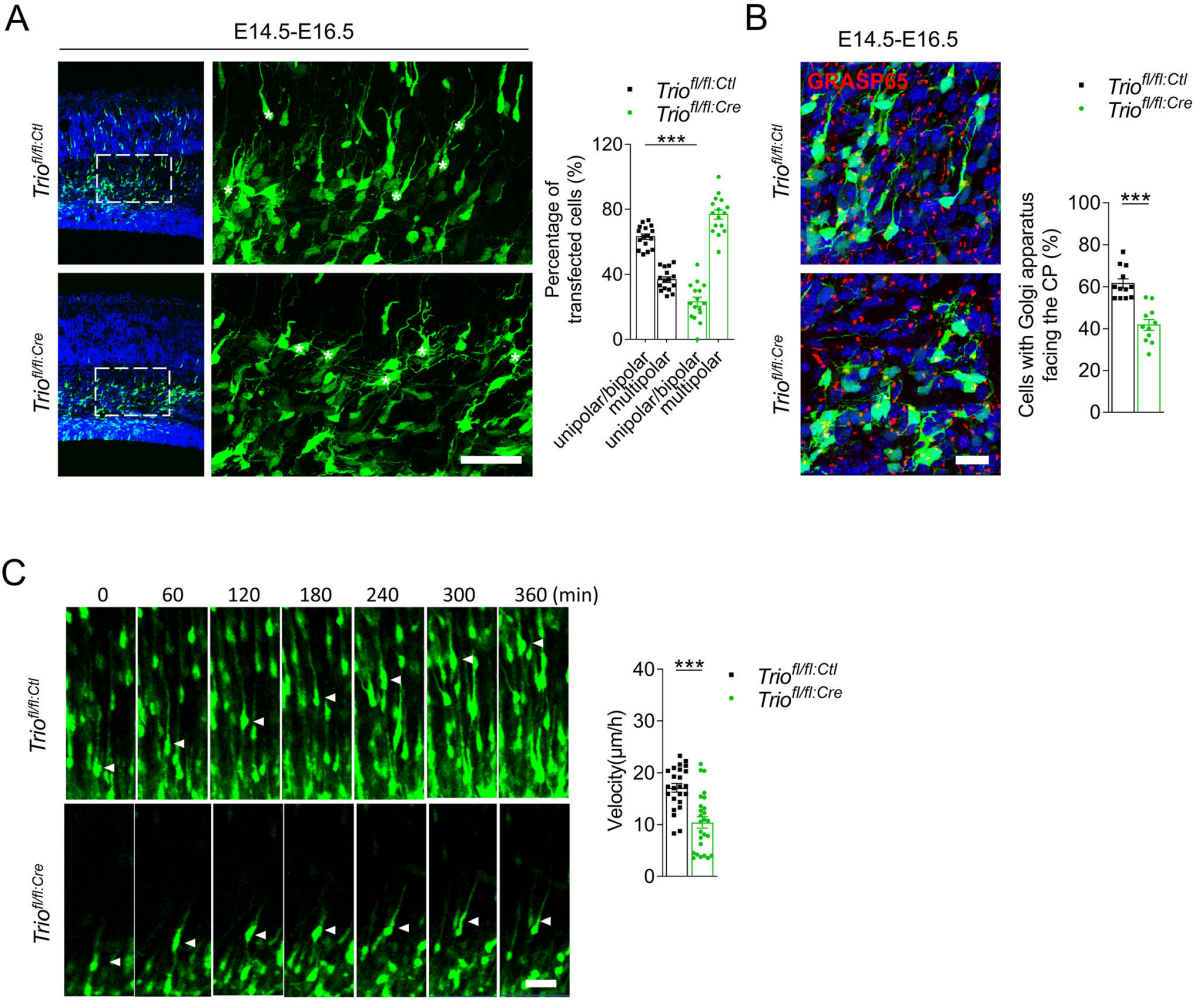
Trio ablation leads to abnormal morphological transition and migratory behavior of cortical projection neurons.** A** Left panels, representative images of GFP^+^ neuronal morphology (white asterisks) at E16.5 in *Trio*^*fl/fl*^ brains electroporated with either *pcAGGS*-*Cre-IRES-GFP* or a control vector at E14.5 (dotted boxes over the IZ are magnified on right) Scale bars, 50 μm. Right panel, percentages of unipolar/bipolar and multipolar neurons in each condition (*n* = 16 slices from 3 *Trio*^*fl/fl:Ctl*^ mice and *n* = 16 slices from 3 *Trio*^*fl/fl:Cre*^ mice).. **B** Representative images and quantification of the proportion of neurons with GRASP65 facing the CP in the IZ 2 days after E14.5 electroporation in *Trio*^*fl/fl:Ctl*^ and *Trio*^*fl/fl:Cre*^ cells (*n* = 12 slices from 3 *Trio*^*fl/fl:Ctl*^ mice and *n* = 11 slices from 3 *Trio*^*fl/fl:Cre*^ mice). Scale bar, 20 μm. **C** Left panels, representative images of time-lapse recording of migrating neurons in cultured cortical tissue after electroporation with the indicated plasmids. Arrowheads, migrating EGFP^+^ neurons. Scale bar, 20 μm. Right panel, speed of migrating EGFP^+^ neurons in each condition (*n* = 24 *Trio*^*fl/fl:Ctl*^ neurons and *n* = 26 *Trio*^*fl/fl:Cre*^ neurons). Histograms show the mean ± SEM. *P < 0.05, **P < 0.01 ***P < 0.001, Student’s *t* test.

Both the morphological transition from MP-to-BP and the correct migratory orientation in the terminal location are important for the migration of late-born neurons. *Trio*^*fl/fl:Ctl*^ and *Trio*^*fl/fl:Cre*^ neurons electroporated at E14.5 were analyzed at E16.5 to investigate neuronal morphology in the upper IZ. The proportion of unipolar/bipolar cells was lower in *Trio*^*fl/fl:Cre*^ neurons than in controls (Fig. 2A), suggesting that the MP-to-BP transition is affected by *Trio* ablation. The Golgi apparatus marker GRASP65 was labeled to assess the migratory direction of neurons in the IZ. The proportion of *Trio*^*fl/fl:Cre*^ neurons with the Golgi apparatus located toward the CP decreased in the IZ compared with *Trio*^*fl/fl:Ctl*^ neurons (Fig. 2B), which is in accordance with a defect in the MP-to-BP transition. In addition, time-lapse imaging of cortical slice cultures *ex vivo* was used to examine the migratory behaviors of cortical projection neurons. *Trio*^*fl/fl:Ctl*^ and *Trio*^*fl/fl:Cre*^ slices, in which neuronal progenitors were electroporated at E13.5, were prepared at E15.5 and cultured for 8 h before a time-lapse imaging analysis. Images of the radial migration of projection neurons from the upper IZ to the CP were captured once every hour for a total of 6 h (Fig. 2C). Compared with *Trio*^*fl/fl:Ctl*^ neurons, most *Trio*^*fl/fl:Cre*^ neurons halted in the IZ and failed to move into the CP. In addition, *Trio*^*fl/fl:Cre*^ neurons traveled shorter distances and exhibited a reduced migration speed (Fig. 2C). Immunostaining of the electroporated *Trio*^*fl/fl:Cre*^ and *Trio*^*fl/fl:Ctl*^ brains as early as 1 day after electroporation (E15.5) was used to assess cell-autonomous proliferation and apoptosis. The percentage of EGFP^+^ cells co-stained with Ki67 cells among the total EGFP^+^ cells did not differ between the two groups in the VZ, where the progenitors were actively dividing (Fig. S2A). In addition, no apoptotic EGFP^+^ cells were present, as indicated by the absence of active caspase-3 staining (Fig. S2B). These data suggested that Trio plays a role in the morphological transition of neurons and the migration velocity, but not in proliferation and apoptosis, in the early stages of the radial migration to the CP.

### Trio Affects Neuronal Polarization by Regulating F-actin Polymerization

**Fig. 3:**
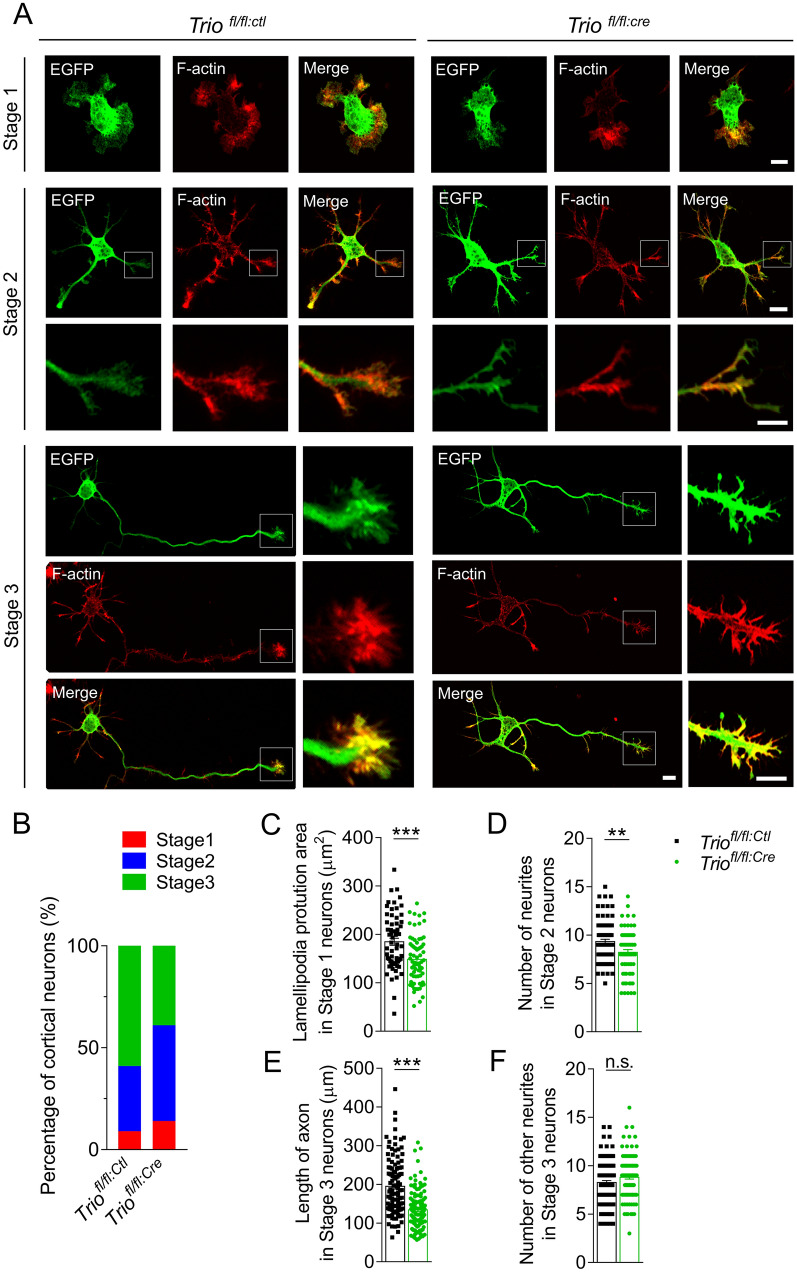
Trio ablation leads to decreased polymerization of F-actin and abnormal neuronal polarization.** A** Representative images showing polymerization of F-actin in electroporated *Trio*^*fl/fl:Cre*^ and *Trio*^*fl/fl:Ctl*^ neurons *in vitro* at stages 1, 2, and 3. Scale bars, 10 μm (stage 1, stage 2 upper panel, and stage 3 left panel) and 5 μm (stage 2 lower panel and stage 3 right panel). **B** Percentages of *Trio*^*fl/fl:Cre*^ and *Trio*^*fl/fl:Ctl*^ neurons at different stages. **C** Area of lamellipodia protrusion in stage 1 neurons in the two groups. **D** Number of neurites in stage 2 neurons in the two groups. **E, F** Length of axons (**E**) and the number of other neurites (**F**) in stage3 *Trio*^*fl/fl:Cre*^ and *Trio*^*fl/fl:Ctl*^ neurons. Histograms show the mean ± SEM. *P < 0.05, **P < 0.01, ***P < 0.001; n.s., no significant difference, Student’s *t* test.

The neurons from *Trio*-deleted and control cortex were extracted immediately after electroporation at E14.5 and cultured for 3 days to further explore the mechanisms induced by *Trio* deletion to regulate the MP-to-BP transition. During the neuronal polarization *in vitro*, nonpolar stage 1 neurons began to grow multiple short neurites to stage 2. A multipolar stage 2 neuron allowed one of its neurites to rapidly grow and become an axon-forming stage 3 neuron [27, 28]. The structural organization and dynamics of the growth cone are essential for neuronal polarization and migration. Thus, we evaluated the distribution of filamentous actin (F-actin) in the growth cones. The distribution of F-actin in the cellular extensions was limited in stage 1 *Trio*^*fl/fl:Cre*^ neurons compared with the control. The polymerized F-actin level decreased in the extended neurites of stage 2 *Trio*^*fl/fl:Cre*^ neurons. The decreased F-actin content resulted in shorter axon length at the polarized growth cone in the stage 3 *Trio*^*fl/fl:Cre*^ neuron (Fig. 3A). In addition, fewer stage 3 neurons and increased proportions of stage 1 and stage 2 neurons were observed in the *Trio*^*fl/fl:Cre*^ group compared with the control (Fig. 3B). The average area of lamellipodia protrusion in stage 1 neurons was significantly smaller in *Trio*^*fl/fl:Cre*^ neurons than in control (Fig. 3C). Stage 2 *Trio*^*fl/fl:Cre*^ neurons also had fewer neurites compared with controls (Fig. 3D). Decreased axon length (Fig. 3E) but a similar number of other neurites (Fig. 3F) were found in stage 3 *Trio*^*fl/fl:Cre*^ neurons compared with controls. These results indicated that *Trio* ablation leads to decreased F-actin polymerization in the growth cone and then abnormal neuronal polarization.

### Trio Regulates Myosin X-dependent Cell Surface Localization of N-cadherin to Promote Neural Adhesion to Radial Glial Fibers

**Fig. 4:**
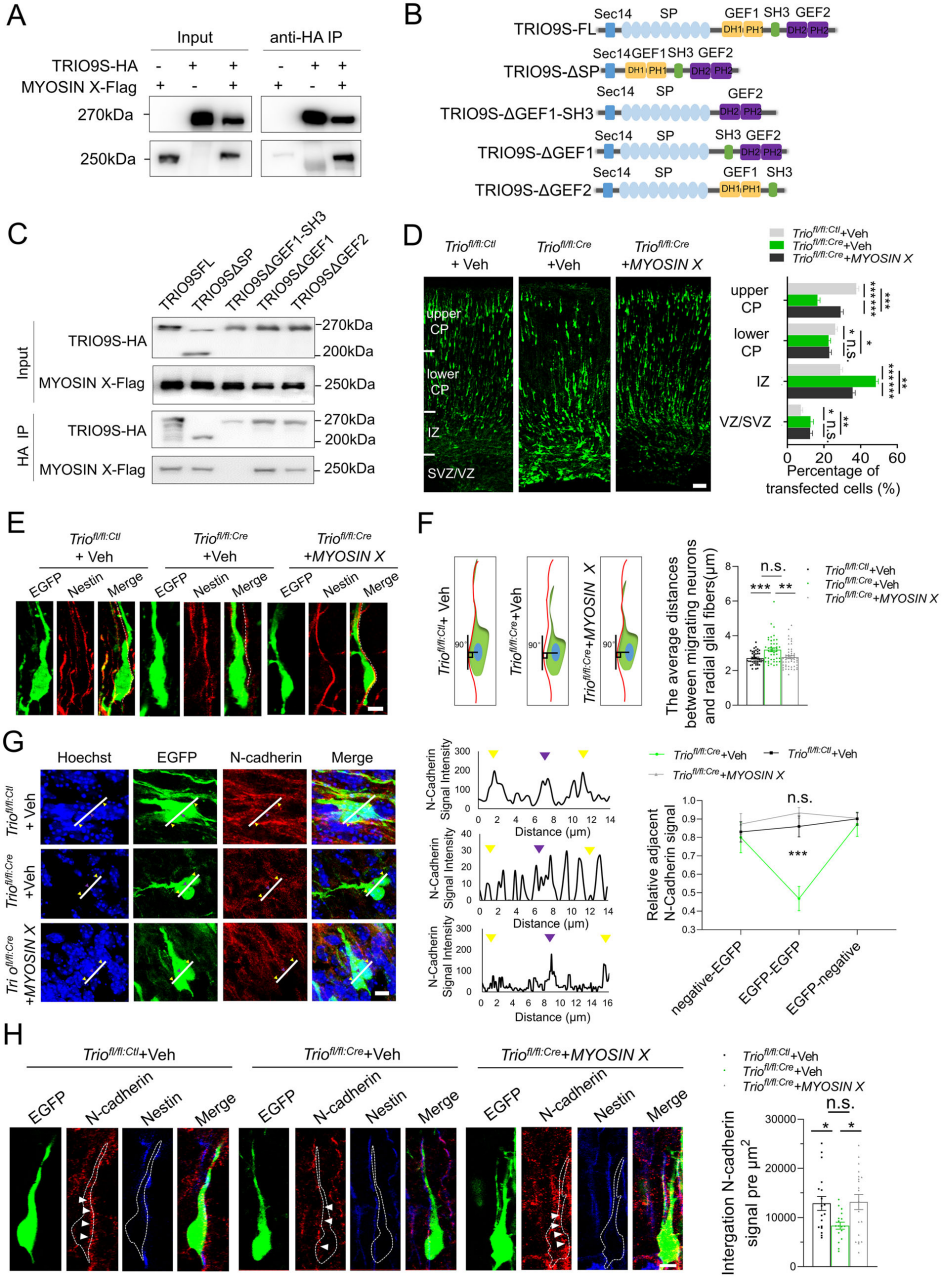
Trio promotes radial migration through Myosin X–dependent N-cadherin neuronal adhesion.** A** Interaction between exogenous TRIO9S and MYOSIN in lysates of HEK293T cells transfected with the indicated plasmids and immunoprecipitated. **B** Schematic of the TRIO9S domains and truncated mutants. **C** Immunoblots of co-immunoprecipitants by the full-length and numerous TRIO9S domain deletions with Myosin X. **D** Left panels, representative images of E17.5 embryonic cerebral cortex electroporated with indicated plasmids at E14.5. Scale bar, 50 μm. Right panel, distribution of EGFP^+^ cells in the VZ/SVZ, IZ, lower CP, and upper CP in each condition. Histograms show the mean ± SEM from at least 12 slices from 3 brains. **E** Representative images of EGFP^+^ cells electroporated with indicated plasmids and Nestin^+^ radial glial fibers (red). Scale bar, 5 μm. **F** Schematic of quantitative analysis (left panel), and the average distance between the center of the soma of the leading process and the Nestin^+^ radial glial fiber in each condition (right panel). Histograms show the mean ± SEM from at least 30 cells from 3 mice for each group. **G** Left panel, E17.5 cortical sections electroporated at E14.5 with indicated plasmids immunostained with N-cadherin antibody. Scale bar, 5 μm. Middle panels, representative expression profiles of N-cadherin measured across the cell bodies of paired EGFP^+^ neurons. Right panel, the relative adjacent N-cadherin signal quantified at the first EGFP^-^/EGFP^+^ cell membrane/border (yellow arrowheads, as negative-EGFP), intermediate EGFP^+^/EGFP^+^ cell membrane/border (purple arrowheads, as EGFP-EGFP), and last EGFP^+^/EGFP^−^ cell membrane/border (yellow arrowheads, as EGFP-negative) of two adjacent EGFP^+^ cells for each condition. The first EGFP^-^/EGFP^+^ cell membrane/border adjacent N-cadherin signal was used for normalization. **H** N-cadherin signal density at the contact sites of migrating EGFP^+^ neurons against radial fibers under every condition. Histograms show the mean ± SEM from at least 20 neurons from 3 brains for each group. *P < 0.05, **P < 0.01, ***P < 0.001, n.s., no significant difference, Student’s *t* test.

Alternative splicing of *Trio* gives rise to numerous isoforms [29]. The two isoforms without a C-terminal kinase domain, TRIO9S and TRIO9L, are considered the dominant isoforms expressed in the brain [30–32]. We investigated Trio interaction partners using mass spectrometry to explore other possible mechanisms underlying Trio-mediated radial migration, especially the decreased migration rate in *Trio*^*fl/fl:Cre*^ BP-shaped neurons. Among many Trio interaction candidates, we focused on the membrane delivery protein Myosin X, as it is involved in radial migration and signal transduction. TRIO9S co-immunoprecipitated with MYOSIN X in HEK293T cells (Fig. 4A). TRIO9S was constructed with seven spectrin repeat domain (SP) deletion (TRIO9SΔSP), GEF1 and SH3 domain deletion (TRIO9SΔGEF1-SH3), GEF1 domain deletion (TRIO9SΔGEF1), or GEF2 domain deletion (TRIO9SΔGEF2; Fig. 4B). The binding affinities of the numerous TRIO9S domain truncations were used to determine the region of TRIO9S necessary for interaction with MYOSIN X. We found that the SH3 domain, but not the GEF1, GEF2, or SP domain, was important for the interaction of TRIO9S with MYOSIN X (Fig. 4C). We co-electroporated MYOSIN X and Cre into the developing *Trio*^*fl/fl*^ cortex to determine whether Myosin X overexpression rescued the migration defect caused by the loss of Trio. Myosin X overexpression partly rescued the migration defects in Trio-deleted neurons (Fig. 4D). During radial migration to the CP, the disruption of the radial glial fibers or the attachment of migrating neurons to the fiber led to abnormal neuronal migration. Nestin immunofluorescence revealed no abnormal morphology of radial glial fibers in *Trio*^*fl/fl:Cre*^ slices (Fig. S3A, B). The attachment of electroporated BP-shaped neurons to Nestin-positive (Nestin^+^) radial glial fibers in the upper IZ and lower CP regions was analyzed (Fig. 4E). The vertical distance between the center of the soma of EGFP^+^ BP neurons and the Nestin^+^ radial glial fibers was measured to assess the characteristics of the neuronal adhesion. Trio deficiency led to an increase in the distance between the center of the soma of BP neurons and the radial glial fibers compared with that measured in control cells. The overexpression of MYOSIN X rescued the defect, and the distance was similar to that measured in controls (Fig. 4F). Myosin X regulates the subcellular distribution of N-cadherin, promoting N-cadherin trafficking from the Golgi apparatus and endosomal sorting of vesicles to the plasma membrane [33]. Immunofluorescent staining revealed N-cadherin expression on the plasma membrane of E17.5 neurons in each group. The N-cadherin levels on the plasma membrane located between two adjacent EGFP^+^ neurons were quantified and normalized to that on the plasma membrane between adjacent EGFP-negative neurons. Strikingly, the expression of N-cadherin at the cell borders between two adjacent EGFP^+^ neurons was reduced in Trio-ablated neurons compared with the control group. The overexpression of Myosin X rescued the decreased membrane expression of N-cadherin (Fig. 4G). We also found the decreased density of N-cadherin at the contact sites of migrating neurons and radial fibers in the *Trio*^*fl/fl:Cre*^ neurons, and Myosin X overexpression rescued the abnormal distribution of N-cadherin in the *Trio*^*fl/fl:Cre*^ neurons (Fig. 4H). These data suggested that the Trio−Myosin X complexes promote neural adhesion and migration along radial glial fibers through N-cadherin expression at the cell surface of cortical projection neurons.

### Overexpression of RAC1 and RHOA Rescues the Radial Migration Impairment by the Loss of Trio

**Fig. 5:**
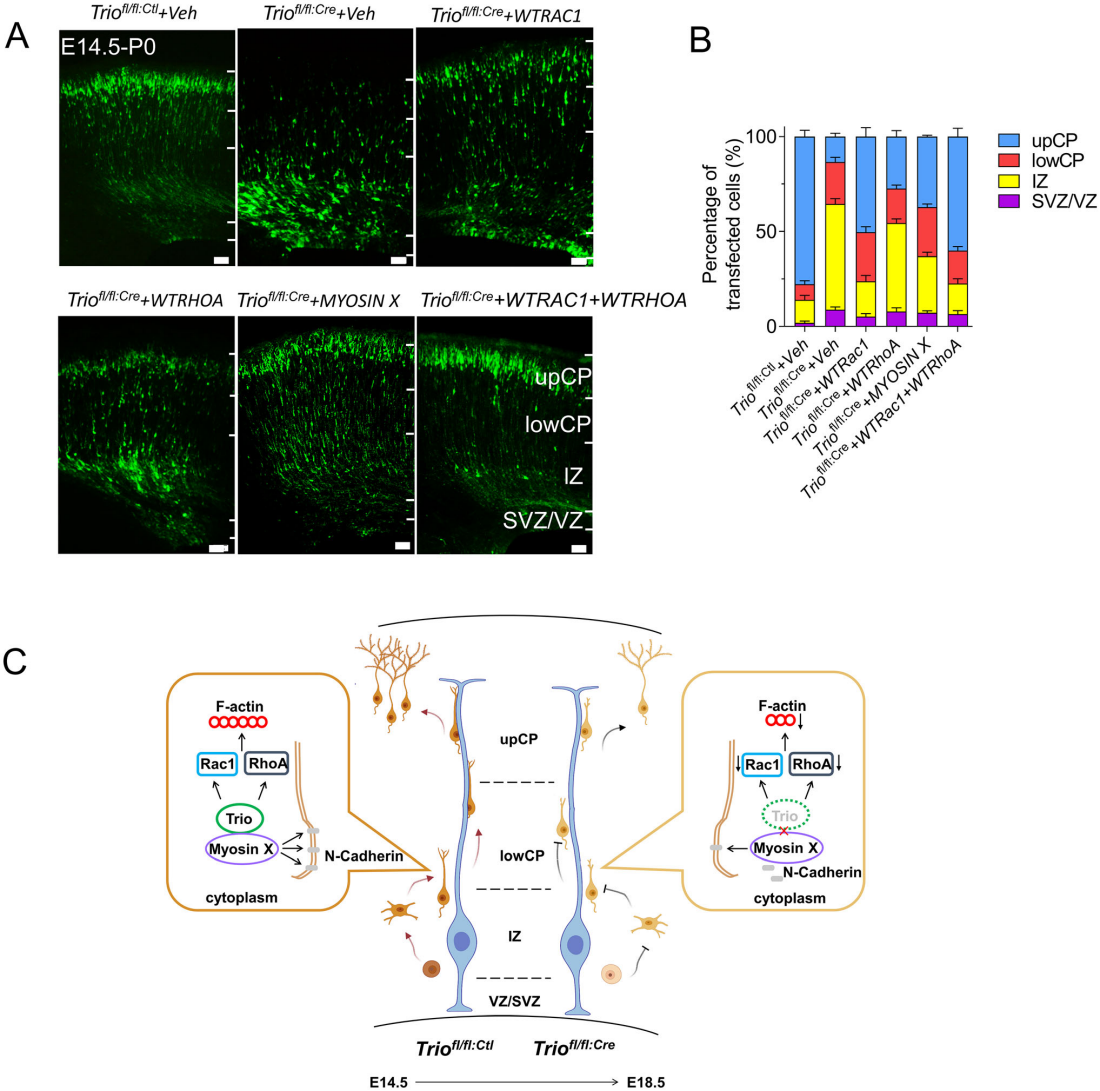
Defects of neuronal migration are rescued by overexpressed RAC1 and ROHA in *Trio*-ablated cells.** A** Co-electroporation of p*cAGGS-Cre-IRES-EGFP* with RAC1, RHOA, MYOSIN X, RAC1, and RhoA into E14.5 *Trio*^*fl/fl*^ brains at E18.5; the p*cAGGS-IRES-EGFP* plasmid serves as control. Scale bars, 50 μm. **B** Relative distributions (percentages) of EGFP-expressing cells in the VZ/SVZ, IZ, lower CP, and upper CP under each condition as in (**A**). Histogram shows the mean ± SEM from at least 12 slices from 3 brains. **C** Model of the role of *Trio* in the migration of cortical projection neurons. *Trio* ablation affects the multipolar-bipolar transition by regulating F-actin dynamics and the radial glial fiber relative migration by locating cell membrane N-cadherin in late-born neurons.

As shown in Fig. 4D, Myosin X overexpression in *Trio*-deleted neurons had a limited effect during radial migration, suggesting that Trio regulates radial migration through other collaborative pathways. The levels of active Rac1, Cdc42, and RhoA, as effectors of Trio, were significantly reduced in the *Trio*-deficient brain at P0.5 [14]. Rac1 and RhoA were overexpressed in *Trio*-deleted neurons to explore the effect of downstream GTPase on *Trio*-mediated radial migration (Fig. 5A). Similar to Myosin X overexpression, Rac1 overexpression promoted the migration of *Trio*-deleted neurons from the IZ to the CP. Moreover, the proportion of *Trio*^*fl/fl:Cre*^ neurons in the upper CP increased upon the expression of RAC1. RhoA overexpression increased the migration of *Trio*^*fl/fl:Cre*^ neurons from the IZ to the CP. Simultaneously, more neurons migrated from the lower CP to the upper CP. In addition, co-overexpressed Rac1 and RhoA effectively rescued the migratory defects of *Trio*-ablated neurons (Fig. 5B). The CA forms of PAK1 and ROCK [34, 35], the Rac1 and RhoA corresponding effector kinases, were electroporated into *Trio*-deleted neurons to investigate further the effects of decreased Rho GTPase activity caused by *Trio* deletion. Although CA-PAK1 overexpression in *Trio*-deleted neurons failed to rescue the migratory defects, CA-ROCK overexpression promoted more *Trio*-deleted neurons from stagnation in the IZ into the CP (Fig. S4). These results suggested that the reduced activation of Rac1 inhibits both the MP-to-BP transition and radial glial-dependent migration, while the reduced activation of RhoA restricts the migration of cells located in the lower CP into the upper CP in Trio-deficient neurons.

## Discussion

In this study, we investigated the role of Trio in cortical radial migration. Our results revealed that Trio was essentially involved in neuronal morphogenesis and the MP-to-BP transition of late-born PNs. Furthermore, Trio interacted with Myosin X through its N-terminal SH3 domain, which regulated the membrane location of N-cadherin and was critical for the neuronal adhesion and migration along radial glial fibers. As Trio is a Rho GEF, the activation of Rac1 and RhoA mediated by the GEF1 and GEF2 domains played independent and synergistic roles in the radial migration of PNs. These findings suggested the vital role of Trio-related signaling in the cortical radial migration of PNs.

The homozygous *Trio* knockout in mice is lethal during embryonic development, as previously reported [36]. Thus, *in utero* electroporation and *Emx1*-mediated conditional *Trio* deletion were used to study the radial migration during embryonic development. Our results suggested that *Trio* deletion affected both the MP-to-BP transition and glial-guided migration in late-born PNs but not the migration of early-born neurons. In addition, *Trio* ablation in early postmitotic PNs resulted in neuronal migration deficits similar to *Trio* ablation in VZ neural progenitors, indicating the importance of *Trio* in the migration of postmitotic neurons. However, the role of *Trio* in neural progenitors during development cannot be ignored and deserves further study, although the deletion of *Trio* has no effect on proliferation or apoptosis.

Similar to the *Trio*-ablation-induced radial migration deficits reported in this study, previous studies have demonstrated that Rac1 knockdown or inhibition of its activity with N17-Rac1 blocks radial migration and disrupts the formation of the leading process [37–39]. Meanwhile, the overexpression of wild-type RAC1 facilitated the migration of *Trio*-deleted neurons from the IZ to the lower CP, further highlighting the vital role of Rac1 in radial migration. However, in accordance with the effect of CA-Rac1 overexpression on neuronal migration [38, 39], the overexpression of a constitutively active form of PAK1, the effector kinase of active Rac1 for regulating F-actin dynamics [40–43], showed a poorer migratory behavior in *Trio*-deleted neurons, indicating that the appropriate activity of Rac1-Pak1 signaling is vital for neuronal polarity in radial migration. However, the overexpression of wild-type RhoA or a constitutively active form of ROCK in *Trio*-ablated neurons in the present study facilitated the migration of more IZ-trapped cells to the CP, which is inconsistent with previous findings [44–47]. This might be attributed to the different genetic context of *Trio* deletion in this study, in which the activities of Rac1 and RhoA were affected simultaneously. Comprehensively, our findings provided evidence that Trio facilitates Rac1 and RhoA signaling to maintain the morphological transition, migration, and adhesion of cortical projection neurons. Trio GEF1 and GEF2 might have distinct and cooperative actions on their Rho GTPase effectors to promote the radial migration of PNs.

Endocytosis and recycling of N-cadherin in migratory neurons promote the interaction between neurons and radial glial fibers in neuronal radial locomotion [48]. The suppression of Myosin X *via in utero* electroporation led to the migration of a reduced number of neurons in the CP and the accumulation of neurons in the IZ due to abnormal N-cadherin distribution into the Golgi apparatus and endosomal sorting vesicles [33]. Previous studies demonstrated that both Myosin X and Trio interact with DCC (deleted in colorectal cancer), a receptor of Netrin-1 [10, 49], and the Rac1 activation elicited by Netrin-1 is lost in the Trio-deficient cortex [10]. Otherwise, RhoA and Rock promote Myosin activation by phosphorylating the Myosin light chain to regulate cell polarity, cell migration, and cell‐cell adhesion junctions in some cell types [50–54]. However, the relationship between Myosin X and Trio-meditated signaling is largely unknown. In this study, we found that Trio was associated with Myosin X. The overexpression of Myosin X facilitated the migration of *Trio*-deleted neurons into the upper CP and a tight attachment to radial glial fibers through regulating the membrane localization of N-cadherin, indicating that Trio-mediated Rac1 and RhoA signaling is involved in the Myosin X-mediated membrane location of N-cadherin in radial migration.

Taken together, our results provide evidence for a novel mechanism of N-cadherin cell surface expression regulated by the Trio-Myosin X complex. The GEF1 and GEF2 domains of Trio and the related signaling have distinct and cooperative actions in regulating radial migration by activating their Rho GTPase effectors.

## Supplementary Information

Below is the link to the electronic supplementary material.

Supplementary file1 (PDF 1344 kb)
