# Supplementary material for: RhoGEF Trio Regulates Radial Migration of Projection Neurons via Its Distinct Domains
Source: Neurosci Bull. 2021 Dec 16;38(3):249–62. doi: 10.1007/s12264-021-00804-7 (PMC8975900; doi:10.1007/s12264-021-00804-7)
Supplement: Supplementary file 1 — Supplementary file1 (PDF 1344 kb) [file 12264_2021_804_MOESM1_ESM.pdf]

Supplementary Figure legends

Figure S1

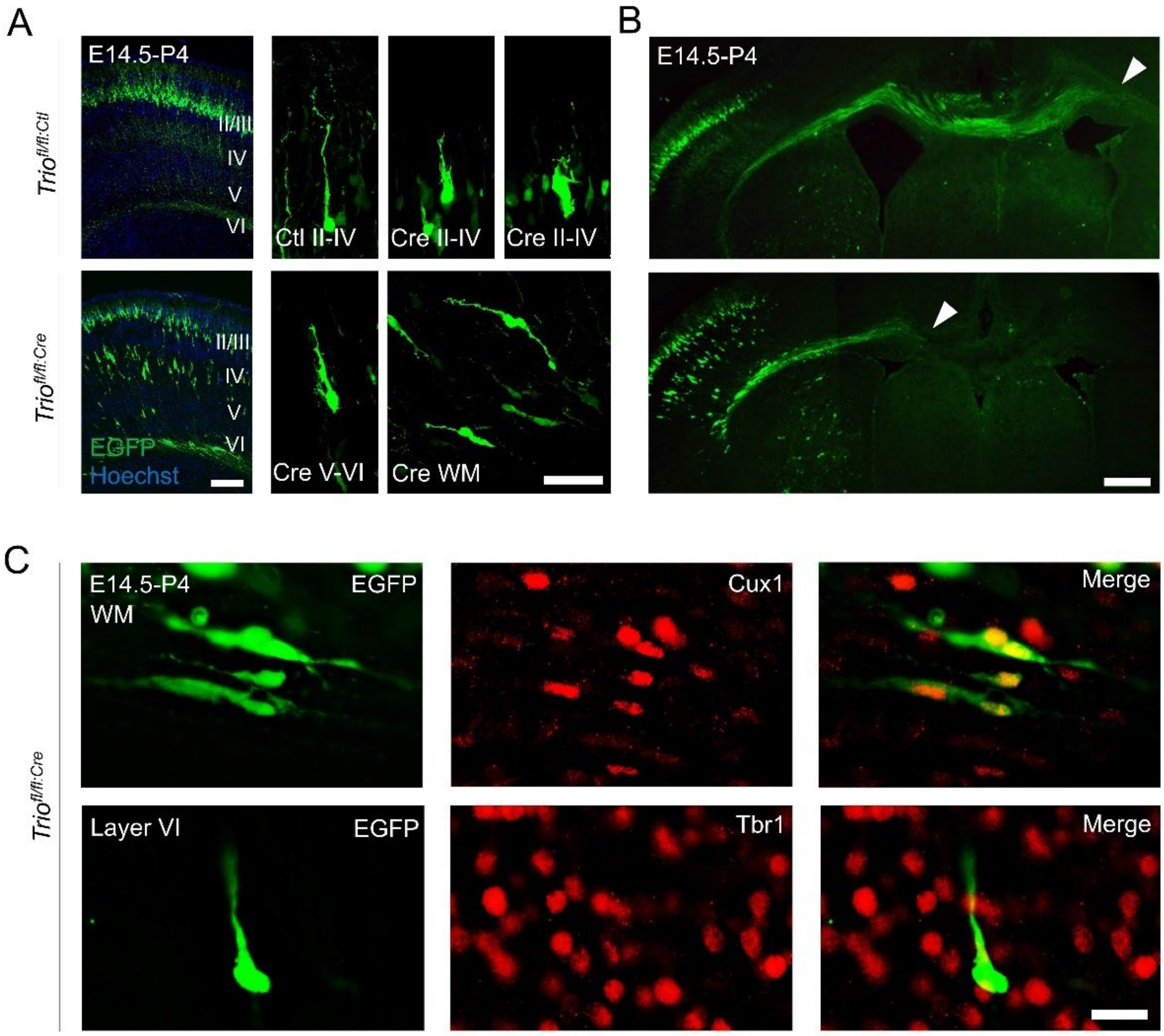

Fig S1. Morphology at P4 of cortical projection neurons electroporated with *Trio*$^{fl/fl:Ctl}$ and *Trio*$^{fl/fl:Cre}$ on E14.5. A Representative images of neural morphology and neurites in layers II–IV of *Trio*$^{fl/fl:Cre}$ and *Trio*$^{fl/fl:Ctl}$ neurons. *Trio*$^{fl/fl:Cre}$ neurons are detained in layers V–VI and subcortical WM. Scale bars, 200 µm (left panels) and 50 µm (right panels). **B** Axons of ipsilateral electroporated *Trio*$^{fl/fl:Ctl}$ and *Trio*$^{fl/fl:Cre}$ cortical projection neurons extend to the contralateral side (arrowheads). Scale bar, 400 µm. **C** EGFP^{+} trapped cells are Cux1^{+} in the WM, but not Tbr1^{+} in layer VI *Trio*$^{fl/fl:Cre}$ neurons. Scale bar, 20 µm.

Figure S2

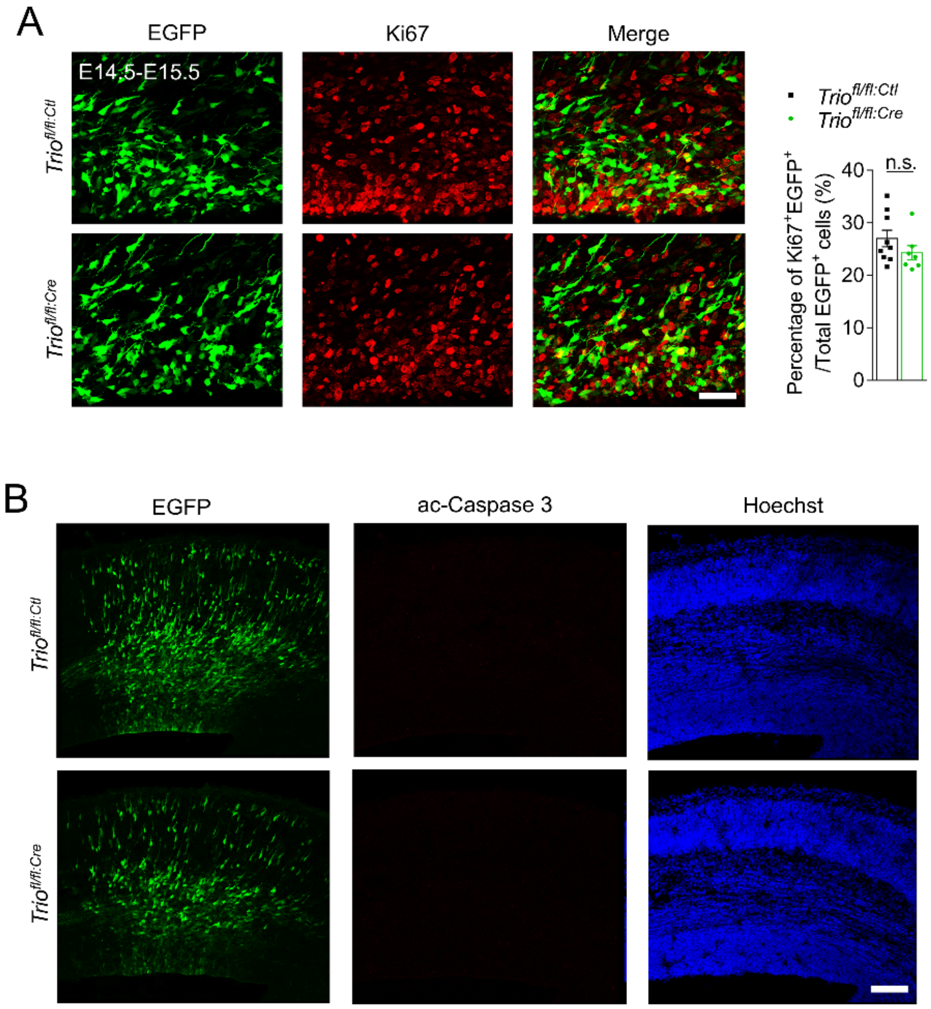

Fig S2. *Trio* deletion affects the radial migration of postmitotic neurons but not the proliferation or apoptosis of progenitors. A Representative images (left) and measurement of proliferation (right) by Ki67 immunostaining of *Trio*$^{fl/fl:Cre}$ and *Trio*$^{fl/fl:Ctl}$ progenitor cells 1 day after E14.5 electroporation. Scale bar, 50 µm. Histogram shows the mean ± SEM. n.s., no significant difference, Student's *t* test. **B** Staining of cleaved caspase-3 for apoptosis in *Trio*$^{fl/fl:Cre}$ and *Trio*$^{fl/fl:Ctl}$ embryonic cortex. Scale bar, 100 µm.

Figure S3

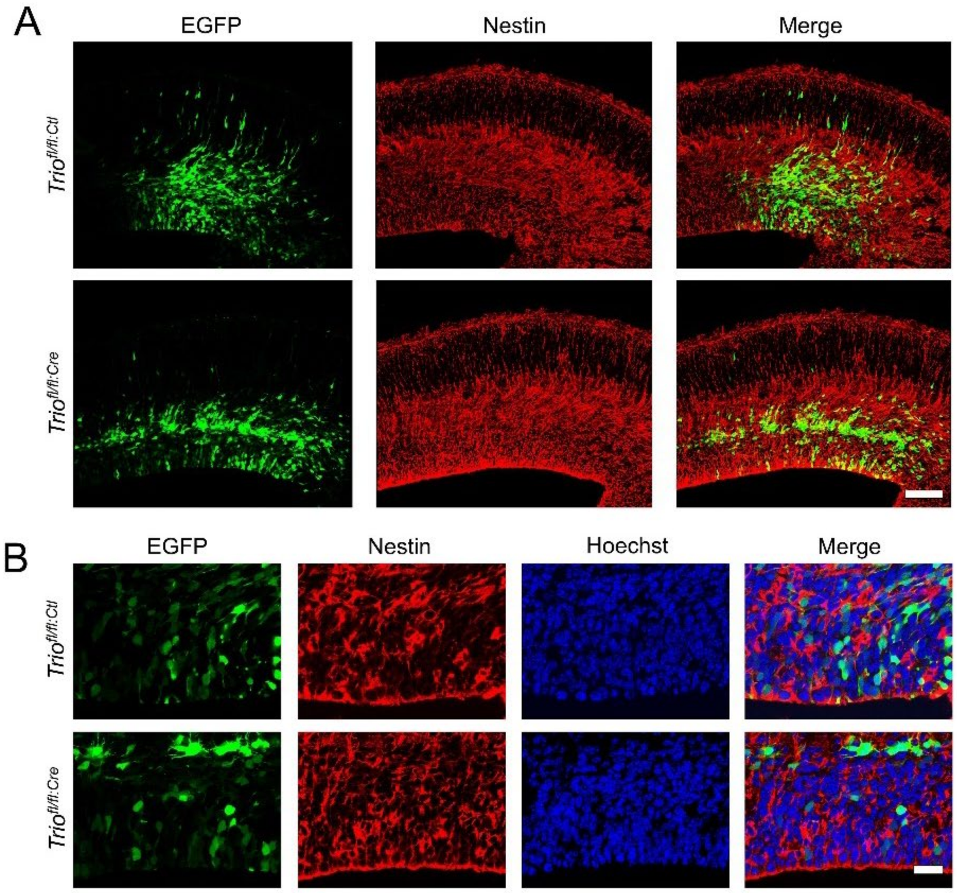

Fig S3. Normal radial glial scaffold in *Trio*-deleted cortical slices. A Nestin-immunostained radial glial cells in $Trio^{fl/fl:Cre}$ and $Trio^{fl/fl:Ctl}$ embryonic cortex 2 days after E14.5 electroporation. Scale bar, 100 µm. **B** Magnification of the VZ/SVZ of the two groups. Scale bar, 25 µm.

Figure S4

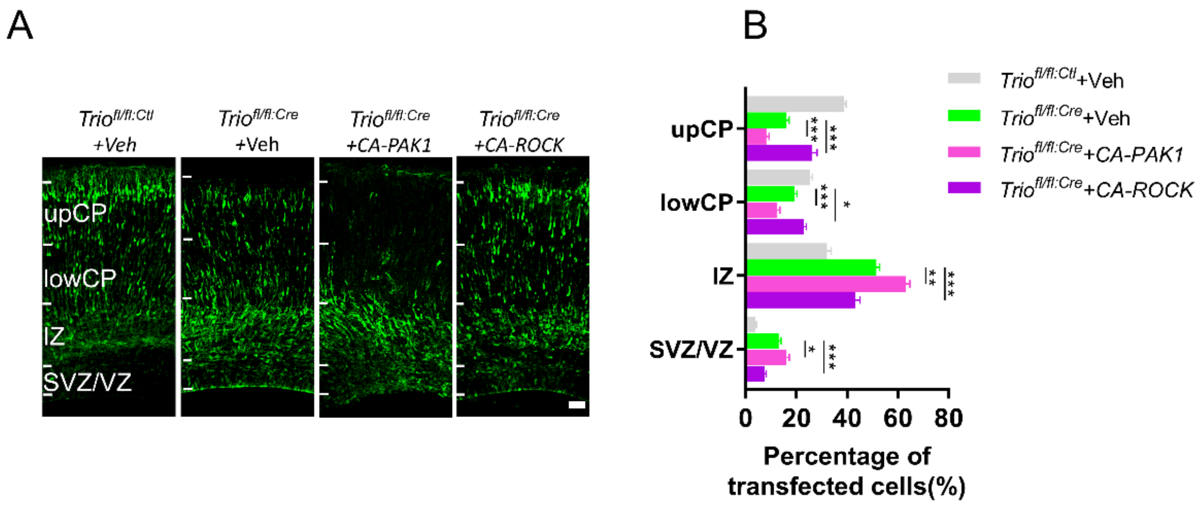

Fig S4. Overexpression of the constitutively-active form of PAK1 and ROCK in migratory *Trio*-ablated neurons. A Representative images from *Trio*$^{fl/fl}$ brains electroporated with indicated plasmids at E14.5 and analyzed at E17.5. Scale bar, 50 µm. **B** The distribution (percentage) of EGFP$^+$ cells in the VZ/SVZ, IZ, lower CP, and upper CP for each condition. Mean ± SEM from at least 12 slices from 3 brains. **P* <0.05, ***P* <0.01, ****P* <0.001, Student's *t* test.
